# Supplementary material for: Comparative Analysis of miRNA-mRNA Regulation in the Testes of Gobiocypris rarus following 17α-Methyltestosterone Exposure
Source: Int J Mol Sci. 2023 Feb 20;24(4):4239. doi: 10.3390/ijms24044239 (PMC9968023; doi:10.3390/ijms24044239)
Supplement: Supplementary file 1 [file ijms-24-04239-s001.zip › Supplementary Figure and Table legends.pdf]

Fig S1 STEM analysis of different profiles in the RNA-seq data and the enriched KEGG pathways. (a) numbers of different profiles based on Q-value (red showed <0.05) and enriched KEGG pathways. (b), the top 20 enriched KEGG pathways by the comparisons among four treatment groups by STEM analysis. (c), the enriched KEGG pathways by STEM analysis. The number in the grid is the p-value/Q-value; "NA" means that the pathway is not enriched for the trend. The darker the lattice color, the more significant the pathway enrichment.

Fig S2 Network heatmap of RNA-miRNA and black, blue and . (a) heat map drawn from clusters of the module genes. Each row and column represents a gene, and the darker the color of each point (white→yellow→red) represents the stronger connectivity between the two genes corresponding to the row and column. P-value was calculated by student's t-test. The smaller the p-value, the more significant the correlation between the gene and module. miRNA-target gene interaction network revealed the highest occurrence in the black (disease-related), blue (metabolism) and turquoise (oxidative and protein ubiquitin) modules using WGCNA analysis. The WGCNA result enriched in top 20 KEGG pathways in (b) black, (c) blue and (d) turquoise modules.

Fig S3 The predicted binding sites of miRNA to mRNA. miR-122-x and miR-574-x to the putative binding site in the 3′-UTR of the *faxdc2* mRNA; miR-430-y, lin-4-x and miR-7-y to the putative binding site in the 3′-UTR of the *inhbb* mRNA, and miR-217-x to the putative binding site in the 3′-UTR of the *ihhb* mRNA.

Table S1 Primer sequences for qRT-PCR. The original sequence was acquired from the *G. rarus* transcriptome database compiled in the present study. Primers were designed based on the original sequence. *soat2* (sterol O-acyltransferase 2-like isoform X1, steroid biosynthesis and lipid metabolism), *inhbb* (inhibin beta B chain-like, cytokine-cytokine receptor interaction), *ihhb* (indian hedgehog B protein-like isoform X1, hedgehog signaling pathway), *gatm* (glycine amidinotransferase, arginine and proline metabolism), *faxdc2* (fatty acid hydroxylase domain-containing protein 2, biosynthesis of antibiotics), *ebp* (3βhydroxysteroid-Δ8,7-isomerase,

biosynthesis of secondary metabolites) and *cyp1a1* (cytochrome P450 1a, retinol metabolism) has been selected for qRT-PCR verification. The miRNAs' name were as follows: *miR-122-x, miR-574-x, miR-430-y, miR-217-x, lin-4-x* and *miR-7-y*.

Table S2 Quality of sequencing data (*n*=3).

Table S3 Statistical analysis of DEGs and DEMs (*n*=3). In the comparison of 25 ng L^{-1} 17MT group with the controls, 59 DEGs (including 26 upregulated and 33 downregulated genes) were identified. In the comparison of 25 ng L^{-1} 17MT group with the controls, 49 of the total DEMs (including 15 upregulated and 34 downregulated genes) were identified. Table description: (1) Pair: control verus experimental group name; (2) Up: the number of upregulated genes with significant differences; (3) Down: the number of downregulated genes with significant diffevel: novel miRNAs.

Table S4 The mRNA profile in the comparison by STEM analysis.

Table S5 The significant top 5 KEGG pathways gathered according to the comparison between different concentrations of 17MT addition. 35 hits have been matched in metabolic KEGG pathways in 25 ng/L 17MT groups when compared with controls.

Table S6 The selected RNA-miRNA pairs and its involved pathway.

Table S7 The mRNA and miRNA profile in the comparison by STEM analysis.

Table S8 Correlations between miRNAs and mRNAs[a].

Analyses were performed with SPSS Statistics 22.0.

[a] Analysis was carried out using Pearson's correlation coeffificient.
