# Supplementary material for: Comparative Analysis of miRNA-mRNA Regulation in the Testes of Gobiocypris rarus following 17α-Methyltestosterone Exposure
Source: Int J Mol Sci. 2023 Feb 20;24(4):4239. doi: 10.3390/ijms24044239 (PMC9968023; doi:10.3390/ijms24044239)
Supplement: Supplementary file 1 [file ijms-24-04239-s001.zip › Table S1.pdf]

Table S1 Primer sequences for qRT-PCR. The original sequence was acquired from the *G. rarus* transcriptome database compiled in the present study. Primers were designed based on the original sequence. *soat2* (sterol O-acyltransferase 2-like isoform X1, steroid biosynthesis and lipid metabolism), *inhbb* (inhibin beta B chain-like, cytokine-cytokine receptor interaction), *ihhb* (indian hedgehog B protein-like isoform X1, hedgehog signaling pathway), *gatm* (glycine amidinotransferase, arginine and proline metabolism), *faxdc2* (fatty acid hydroxylase domain-containing protein 2, biosynthesis of antibiotics), *ebp* (3βhydroxysteroid-Δ8,7-isomerase, biosynthesis of secondary metabolites) and *cyp1a1* (cytochrome P450 1a, retinol metabolism) has been selected for qRT-PCR verification. The miRNAs' name were as follows: *miR-122-x, miR-574-x, miR-430-y, miR-217-x, lin-4-x* and *miR-7-y*.

| **Gene** | **primer 5'-3'** |
|---|---|
| *soat2* | F: AAGCACCAACAACAGGCACA<br>R: TGACCGTACTCAACTGACCAAAC |
| *inhbb* | F: CTCGGAGTCGTTCTCGTGG<br>R: ACCGTCTGCGTGATGTTCG |
| *ihhb* | F: AGTGGTGGTCAATGGTGTCGT<br>R: CCAGTGCAGCAGTGAGGAATA |
| *gatm* | F: CTCCGACGCCTCTTATTCCA<br>R: CGATACCGAGATTTTCAAACATT |
| *faxdc2* | F: ACTTCACCTCGCCTTGGG<br>R: AACAGCACCGTCTTCGCC |
| *ebp* | F: CGCAACCTGTCTATTCCCAAC<br>R: ACCTTCTCCCTCCGCTCAA |
| *cyp1a1* | F: TGTCGTCTATCTCGTGGCGTAT<br>R: GGTGAAGGGAAGGAAGGAGG |
| *ef1a* | F:ACAAATGCGGTGGAATCG<br>R:TCAAACTTCCAGAGAGCGATA |
| *u6* | GCGCGGCCTGCATTGTACACAC |
| miR-122-x | GCCGTGGAGTGTGACAATGGTGTTTGT |
| *miR-574-x* | CCGGTGAGTGTGTGAGTGTGAGTGTGA |
| *miR-430-y* | GCCGCGTAAGTGCTATTTGTTGGGGTAG |
| *miR-217-x* | CCGGGTACTGCATCAGGAACTGATTGGA |
| *lin-4-x* | CCGCCTCCCTGAGACCCTACTTGTG |
| *miR-7-y* | GCCGGCCAACAAGTCATAGTCTTCCTCA |
