# Supplementary material for: Comparative Analysis of miRNA-mRNA Regulation in the Testes of Gobiocypris rarus following 17α-Methyltestosterone Exposure
Source: Int J Mol Sci. 2023 Feb 20;24(4):4239. doi: 10.3390/ijms24044239 (PMC9968023; doi:10.3390/ijms24044239)
Supplement: Supplementary file 1 [file ijms-24-04239-s001.zip › Table S2.pdf]

Table S2 Quality of sequencing data (*n*=3)

Sample	Genes Num-RNA	Ratio-RNA	total	rRNA	snRNA	snoRNA	tRNA	known mirna num	novel mirna num	miRNA_number	target_gene_number
Con	67300±988	0.92±0.01	841909±252753	6725±1338	615±46	158±7	2378±98	424±43	721±65	1145±32	23850±13
MT25-M	67823±1166	0.92±0.02	725271±228382	6590±591	703±77	159±18	2265±125	408±71	674±34	1082±45	23819±79
MT50-M	67709±551	0.92±0.01	773402±290531	8817±1612	754±59	198±31	2753±72	504±109	734±78	1238±53	23961±70
MT100-M	68633±873	0.93±0.01	720409±266741	8650±277	756±32	150±13	2681±151	414±37	691±100	1105±84	23822±69
