# Supplementary material for: Comparative Analysis of miRNA-mRNA Regulation in the Testes of Gobiocypris rarus following 17α-Methyltestosterone Exposure
Source: Int J Mol Sci. 2023 Feb 20;24(4):4239. doi: 10.3390/ijms24044239 (PMC9968023; doi:10.3390/ijms24044239)
Supplement: Supplementary file 1 [file ijms-24-04239-s001.zip › Table S3.pdf]

Table S3 Statistical analysis of DEGs and DEMs (*n*=3). In the comparison of 25 ng L^{-1} 17MT group with the controls, 59 DEGs (including 26 upregulated and 33 downregulated genes) were identified. In the comparison of 25 ng L^{-1} 17MT group with the controls, 49 of the total DEMs (including 15 upregulated and 34 downregulated genes) were identified. Table description: (1) Pair: control verus experimental group name; (2) Up: the number of upregulated genes with significant differences; (3) Down: the number of downregulated genes with significant diffevel: novel miRNAs.

**Pair**	**DGEs, DEMs/known/novel (Up)**	**DGEs, DEMs/known/novel (Down)**
Con-M-VS-MT25-M	26, 15/4/11	33, 34/7/27
Con-M-VS-MT50-M	52, 25/19/13	25, 41/2/15
Con-M-VS-MT100-M	35, 32/6/19	31, 17/14/27
MT25-M-VS-MT50-M	91, 68/27/41	209, 19/7/12
MT25-M-VS-MT100-M	114, 43/12/31	132, 37/13/24
MT50-M-VS-MT100-M	118, 24/5/19	17, 34/18/16
