# Supplementary material for: Comparative Analysis of miRNA-mRNA Regulation in the Testes of Gobiocypris rarus following 17α-Methyltestosterone Exposure
Source: Int J Mol Sci. 2023 Feb 20;24(4):4239. doi: 10.3390/ijms24044239 (PMC9968023; doi:10.3390/ijms24044239)
Supplement: Supplementary file 1 [file ijms-24-04239-s001.zip › Table S5.pdf]

Table S5 The significant top 5 KEGG pathways gathered according to the comparison between different concentrations of 17MT addition. 35 hits have been matched in metabolic KEGG pathways in 25 ng/L 17MT groups when compared with controls.

**Comparison**	**Top 5 pathways**	**hits**	**Q-value**
Con-M-VS-MT25-M	Metabolic pathways	35	9.22E-01
	Cell adhesion molecules (CAMs)	19	6.70E-05
	Neuroactive ligand-receptor interaction	18	2.45E-01
	Phagosome	12	5.29E-02
	Oxidative phosphorylation	10	5.29E-02
Con-M-VS-MT50-M	Metabolic pathways	61	3.93E-01
	Cytokine-cytokine receptor interaction	30	2.88E-07
	Phagosome	22	1.81E-04
	Biosynthesis of secondary metabolites	19	5.46E-01
	Neuroactive ligand-receptor interaction	19	7.71E-01
Con-M-VS-MT100-M	Metabolic pathways	91	0.000174
	Biosynthesis of secondary metabolites	31	0.010379
	Neuroactive ligand-receptor interaction	22	0.693384
	Biosynthesis of antibiotics	22	0.003869
	Phagosome	16	0.078623
MT25-M-VS-MT50-M	Metabolic pathways	107	5.62E-01
	Neuroactive ligand-receptor interaction	45	1.69E-01
	Cell adhesion molecules (CAMs)	44	1.01E-07
	Cytokine-cytokine receptor interaction	44	3.62E-07
	Phagosome	36	8.78E-06
MT25-M-VS-MT100-M	Metabolic pathways	120	1.35E-05
	Biosynthesis of secondary metabolites	42	3.06E-03
	Neuroactive ligand-receptor interaction	37	1.27E-01
	Biosynthesis of antibiotics	23	2.58E-02
	Calcium signaling pathway	21	1.00E+00
MT50-M-VS-MT100-M	Metabolic pathways	51	9.99E-01
	Cytokine-cytokine receptor interaction	41	3.46E-11
	Neuroactive ligand-receptor interaction	30	2.94E-01
	Calcium signaling pathway	27	2.94E-01
	Cell adhesion molecules (CAMs)	24	4.49E-03
