# Supplementary material for: Comparative Analysis of miRNA-mRNA Regulation in the Testes of Gobiocypris rarus following 17α-Methyltestosterone Exposure
Source: Int J Mol Sci. 2023 Feb 20;24(4):4239. doi: 10.3390/ijms24044239 (PMC9968023; doi:10.3390/ijms24044239)
Supplement: Supplementary file 1 [file ijms-24-04239-s001.zip › Table S6.pdf]

Table S6 The selected RNA-miRNA pairs and its involved pathway.

**Id-Profile15**	**Gene name**	**Gene discription**	**Pathway annotation**	**miRNA pairs for target genes**
UNIGENE0040500	*soat2*	sterol O-acyltransferase 2-like isoform X1	Steroid biosynthesis/Lipid metabolism	bantam-x;miR-1241-y;miR-1609-x;miR-1970-x;miR-218-x;miR-2352-x;miR-322-x;miR-382-y;miR-4049-y;miR-424-x;miR-5350-y;miR-6216-x;miR-7573-x;miR-7794-x;miR-9235-x;novel-m0092-3p;novel-m0140-3p;novel-m0194-5p;novel-m0235-5p;novel-m0665-3p;novel-m0716-3p
UNIGENE0027524	*inhbb*	inhibin beta B chain-like	Cytokine-cytokine receptor interaction	**lin-4-x**;miR-1-x;miR-101-y;miR-106-x;miR-107-x;miR-125-x;miR-1434-x;miR-145-y;miR-152-x;miR-17-x;miR-1711-x;miR-199-x;miR-2012-x;miR-2048-y;miR-2062-x;miR-223-x;miR-2304-y;miR-2314-x;miR-2808-y;miR-293-x;miR-300-y;miR-306-x;miR-3068-y;miR-322-y;miR-329-y;miR-33-y;miR-365-y;miR-371-y;miR-3738-x;miR-376-y;miR-425-x;**miR-430-y**;miR-4324-y;miR-4560-x;miR-457-y;miR-4587-y;miR-4791-x;miR-551-x;miR-6703-y;miR-6739-x;miR-6806-y;miR-6854-x;miR-6952-y;**miR-7-y**;miR-7010-x;miR-7031-x;miR-7093-x;miR-731-x;miR-7390-y;miR-743-y;miR-81-y;miR-8390-x;miR-8457-x;miR-8917-y;miR-9054-x;miR-93-x;novel-m0031-5p;novel-m0042-3p;novel-m0096-5p;novel-m0106-5p;novel-m0130-3p;novel-m0139-5p;novel-m0151-3p;novel-m0192-5p;novel-m0193-5p;novel-m0199-3p;novel-m0232-3p;novel-m0235-5p;novel-m0250-5p;novel-m0387-3p;novel-m0388-3p;novel-m0402-3p;novel-m0417-5p;novel-m0432-5p;novel-m0436-5p;novel-m0442-5p;novel-m0472-3p;novel-m0487-5p;novel-m0512-5p;novel-m0522-3p;novel-m0551-3p;novel-m0582-3p;novel-m0583-3p;novel-m0608-3p;novel-m0609-5p;novel-m0658-3p;novel-m0667-3p;novel-m0731-3p;novel-m0775-5p;novel-m0791-3p;novel-m0802-5p
UNIGENE0024517	*ihhb*	indian hedgehog B protein-like isoform X1	Hedgehog signaling pathway	miR-1175-y;miR-1323-x;miR-1339-x;miR-1779-y;miR-184-x;miR-1841-x;miR-187-x;miR-190-x;miR-199-x;**miR-217-x**;miR-22-x;miR-2227-y;miR-223-x;miR-2496-x;miR-297-x;miR-304-x;miR-3099-y;miR-3191-x;miR-3263-x;miR-3536-x;miR-374-y;miR-3808-x;miR-3847-x;miR-3937-y;miR-4016-x;miR-411-x;miR-411-y;miR-449-x;miR-4587-y;miR-466-x;miR-

				4692-y;miR-4925-y;miR-4946-x;miR-497-x;miR-574-y;miR-580-y;miR-5847-x;miR-5857-x;miR-6542-y;miR-6582-x;miR-6618-x;miR-669-x;miR-6767-x;miR-6948-y;miR-71-x;miR-7420-x;miR-743-y;miR-7669-y;miR-7975-y;miR-8254-x;miR-878-x;miR-9054-x;miR-9077-y;miR-9103-y;miR-9384-y;novel-m0130-3p;novel-m0173-5p;novel-m0192-5p;novel-m0193-5p;novel-m0201-5p;novel-m0228-5p;novel-m0229-3p;novel-m0245-3p;novel-m0314-5p;novel-m0386-3p;novel-m0405-3p;novel-m0415-5p;novel-m0530-3p;novel-m0560-5p;novel-m0561-3p;novel-m0593-3p;novel-m0603-5p;novel-m0604-5p;novel-m0608-3p;novel-m0609-5p;novel-m0613-3p;novel-m0678-5p;novel-m0809-3p;novel-m0859-3p;novel-m0879-3p
UNIGENE0072944	*gatm*	glycine amidinotransferase, mitochondrial	Arginine and proline metabolism	miR-1493-x;miR-2250-y;miR-23-y;miR-3009-y;miR-3095-y;miR-325-y;miR-4048-y;miR-460-y;miR-4732-y;miR-5119-y;miR-5950-y;miR-6613-y;miR-6939-x;miR-7072-x;miR-7186-x;miR-753-y;miR-82-y;miR-8343-x;miR-8384-y;miR-9072-y;miR-9186-y;novel-m0090-5p;novel-m0213-5p;novel-m0450-3p;novel-m0506-5p;novel-m0676-3p
UNIGENE0012321	*faxdc2*	fatty acid hydroxylase domain-containing protein 2	Biosynthesis of antibiotics/Steroid biosynthesis/Lipid metabolism	miR-11-y;miR-1187-y;miR-1193-y;**miR-122-x**;miR-1330-x;miR-136-y;miR-140-x;miR-1910-y;miR-2184-x;miR-235-y;miR-2377-y;miR-2408-y;miR-2496-x;miR-25-y;miR-2540-x;miR-30-y;miR-311-y;miR-313-y;miR-3186-y;miR-3574-x;miR-370-y;miR-4062-y;miR-4296-x;miR-434-y;miR-4526-y;miR-466-x;miR-500-y;miR-502-y;miR-5457-y;**miR-574-x**;miR-669-x;miR-7348-y;miR-7390-y;miR-7420-x;miR-7573-x;miR-7860-y;miR-8094-x;miR-8118-y;miR-8309-y;miR-9032-y;miR-9198-x;miR-92-y;miR-9318-x;miR-9385-x;novel-m0006-5p;novel-m0141-3p;novel-m0225-5p;novel-m0239-3p;novel-m0305-5p;novel-m0328-3p;novel-m0344-3p;novel-m0499-3p;novel-m0504-3p;novel-m0507-5p;novel-m0508-5p;novel-m0572-5p;novel-m0582-3p;novel-m0583-3p;novel-m0651-3p;novel-m0692-3p;novel-m0743-5p;novel-m0791-3p;novel-m0837-3p;novel-m0859-3p
UNIGENE0059946	*ebp*	3-beta-hydroxysteroid-Delta(8),Delta(7)-isomerase	Biosynthesis of secondary metabolites	miR-106-x;miR-130-y;miR-1357-x;miR-17-x;miR-20-x;miR-2116-y;miR-219-y;miR-22-y;miR-3015-y;miR-3919-y;miR-3966-x;miR-452-y;miR-454-y;miR-6325-y;miR-6598-x;miR-6639-x;miR-734-y;miR-7373-x;miR-7552-x;miR-93-x;novel-m0222-3p;novel-m0250-5p;novel-m0253-3p;novel-m0410-5p;novel-m0842-3p;novel-m0871-5p;novel-m0876-3p

UNIGENE0032674	*cyp1a1*	cytochrome P450 1a	Retinol metabolism/Steroid hormone biosynthesis/Lipid metabolism/Metabolism of xenobiotics by cytochrome P450/Xenobiotics biodegradation and metabolism	miR-1273-y;miR-1336-x;miR-1421-x;miR-143-y;miR-148-y;miR-1654-x;miR-17-y;miR-1748-y;miR-1770-y;miR-1907-y;miR-202-y;miR-205-x;miR-212-y;miR-218-y;miR-2187-y;miR-23-y;miR-2356-x;miR-239-x;miR-2540-x;miR-2763-y;miR-277-y;miR-3079-y;miR-3137-x;miR-36-x;miR-3718-x;miR-466-y;miR-4770-y;miR-627-x;miR-6576-x;miR-669-y;miR-6915-x;miR-7461-x;miR-7573-x;miR-7634-x;miR-9087-x;miR-9811-x;novel-m0053-3p;novel-m0135-3p;novel-m0173-5p;novel-m0205-5p;novel-m0229-3p;novel-m0325-5p;novel-m0373-3p;novel-m0415-5p;novel-m0464-3p;novel-m0487-5p;novel-m0507-5p;novel-m0508-5p;novel-m0560-5p;novel-m0629-5p;novel-m0646-3p;novel-m0664-5p;novel-m0787-5p;novel-m0788-5p;novel-m0853-3p;novel-m0882-5p
