# Supplementary material for: Comparative Analysis of miRNA-mRNA Regulation in the Testes of Gobiocypris rarus following 17α-Methyltestosterone Exposure
Source: Int J Mol Sci. 2023 Feb 20;24(4):4239. doi: 10.3390/ijms24044239 (PMC9968023; doi:10.3390/ijms24044239)
Supplement: Supplementary file 1 [file ijms-24-04239-s001.zip › Table S7.pdf]

Table S6 The mRNA and miRNA profile in the comparison by STEM analysis.

**RNA ID-Profile15**	**MT25-M**	**MT50-M**	**MT100-M**	**Note**
cyp2j2	-0.06	1.83	2.21	cytochrome P450 2J2-like
pcyt1b	-0.11	1.48	1.43	choline-phosphate cytidylyltransferase B-like
anpep	-0.63	1.95	2.16	aminopeptidase N-like
ptgds	-0.24	1.11	1.33	prostaglandin-H2 D-isomerase-like
hsd11b1l	-0.03	1.5	1.74	11-beta-hydroxysteroid dehydrogenase-like protein
adh5	-0.14	1.41	1.81	alcohol dehydrogenase
gapdh	-0.36	1.33	1.21	Gapdh protein, partial
spam1	0.18	1.66	2.14	hyaluronidase PH-20-like
acp5	-0.78	0.84	0.81	tartrate-resistant acid phosphatase type 5-like
gstz1	-0.08	1	0.98	glutathione S-transferase zeta
bhmt	-0.19	1.44	1.35	Bhmt protein
gys2	-0.11	1.21	0.84	glycogen [starch] synthase, liver-like
pygl	-0.45	1.32	1.35	glycogen phosphorylase, liver form-like
lipc	-0.32	1.66	2.15	hepatic triacylglycerol lipase isoform X1
agmat	0.01	1.01	1.29	agmatinase, mitochondrial
hao1	-0.3	1.45	1.62	hydroxyacid oxidase 1
pkm	-0.03	1.52	0.81	pyruvate kinase
cbs	0.08	1.12	1.44	cystathionine beta-synthase-like
kynu	0.01	1.92	1.84	kynureninase-like isoform X1
acmsd	-0.23	1.09	1.41	2-amino-3-carboxymuconate-6-semialdehyde decarboxylase-like isoform X1
ftcd	-0.5	1.14	1.3	formimidoyltransferase-cyclodeaminase-like
uox	-0.17	0.92	1.29	uricase-like
msmo1	0.19	1.28	1.4	methylsterol monooxygenase 1 isoform X1
kmo	-0.09	0.82	1.01	kynurenine 3-monooxygenase
ugt2b20	-0.03	1.66	1.66	UDP-glucuronosyltransferase 2B15-like isoform X1
grhpr	-0.21	1.28	1.47	glyoxylate reductase/hydroxypyruvate reductase-like
tdo2a	-0.5	1.32	1.14	Tdo2 protein
hpd	-0.2	1.49	1.78	4-hydroxyphenylpyruvate dioxygenase
ugt2a2	0.25	1.16	1.45	UDP-glucuronosyltransferase 2A2
dpys	0.23	1.74	2.01	dihydropyrimidinase

pah	-0.09	1.42	1.5	phenylalanine-4-hydroxylase
ldhbb	-0.55	1.48	0.99	L-lactate dehydrogenase B-B chain
cyp27a1	0.1	0.88	1.18	sterol 26-hydroxylase, mitochondrial-like
gamt	-0.59	1.01	0.82	guanidinoacetate N-methyltransferase
tat	0.54	2.24	2.25	tyrosine aminotransferase isoform X1
pck1	-0.3	1.03	1.01	phosphoenolpyruvate carboxylase
pla2g12b	-0.29	1.82	1.41	group XIIB secretory phospholipase A2-like protein isoform X1
sds	-0.37	0.79	0.83	serine dehydratase-like
fah	-0.17	0.98	0.81	fumarylacetoacetase-like
anpep	0.48	2.8	2.44	aminopeptidase N-like
agxt2	-0.18	0.81	1.07	alanine--glyoxylate aminotransferase 2, mitochondrial-like isoform X1
scp2	0.15	0.98	1.17	non-specific lipid-transfer protein isoform X2
hgd	-0.18	0.94	1.23	homogentisate 1,2-dioxygenase-like isoform X1
me3	-0.72	0.87	0.88	NADP-dependent malic enzyme-like
aldob	0.03	1.79	1.91	fructose-bisphosphate aldolase B-like
upb1	-0.04	1.18	1.5	beta-ureidopropionase
gpt2l	0.18	1.11	1.24	alanine aminotransferase 2-like isoform X1
hmgcr	-0.18	0.78	0.95	3-hydroxy-3-methylglutaryl-coenzyme A reductase
tdo2b	-0.38	1.17	1.39	tryptophan 2,3-dioxygenase B isoform X2
cda	-0.54	1.38	1.49	cytidine deaminase-like
anpep	0.34	5.6	3.05	aminopeptidase N protein
haao	-0.68	1.19	1.58	3-hydroxyanthranilate 3,4-dioxygenase
**miRNA ID-Profile5**				
mir-9-x	-0.89	0.48	0.36	
mir-7133-y	-2.14	0.05	0.16	
mir-551-y	-1.64	0.02	-0.27	
mir-409-x	-1.78	0.6	-0.64	
mir-3963-x	-4.23	2.5	-0.16	
mir-409-y	-6.19	1.27	-0.9	
mir-335-x	-4.23	3.14	2.04	
mir-4968-y	-5.74	1.12	0.54	
mir-2422-x	-4.59	1.49	1.95	

**miRNA ID-Profile11**				
mir-215-x	-0.42	1.72	0.42	
mir-459-x	-1.73	2.45	0.18	
mir-459-y	-1.33	2.91	-1.67	
mir-4792-x	0	5.96	0	
mir-279-y	0	6.7	0	
mir-1966-x	0	6.14	0	
**miRNA ID-Profile12**				
mir-192-x	-0.3	1.62	0.7	
mir-194-x	-0.33	1.41	0.74	
mir-31-x	-0.25	0.99	0.61	
mir-192-z	0.04	1.65	1.13	
mir-72-x	-0.35	0.9	0.63	
mir-722-y	-0.48	0.98	0.76	
mir-122-y	-0.32	1.01	0.61	
mir-9083-x	-1.25	2.93	1.34	
mir-733-x	-0.53	1.33	1.21	
**miRNA ID-Profile13**				
mir-133-z	0.67	0.11	-0.82	
soat2	-0.71	1.36	1.28	sterol O-acyltransferase 2-like isoform X1
inhbb	-1.08	1.42	1.23	inhibin beta B chain-like
ihhb	-0.14	1.46	1.89	indian hedgehog B protein-like isoform X1
gatm	0.04	1.55	1.36	glycine amidinotransferase, mitochondrial
faxdc2	-0.42	1.21	1.43	fatty acid hydroxylase domain-containing protein 2
ebp	-0.39	1.22	1.72	3-beta-hydroxysteroid-Delta(8),Delta(7)-isomerase
cyp1a1	-0.34	1.66	1.37	cytochrome P450 1a
miR-122-x	-0.41	1.12	1.02	
miR-574-x	-2.08	-0.28	-0.3	
miR-430-y	-0.37	1.07	-0.27	
miR-217-x	-0.91	0.94	-0.45	
lin-4-x	0.56	0.19	-0.72	
miR-7-y	0.27	-0.93	-2.33	
