# Supplementary material for: Comparative Analysis of miRNA-mRNA Regulation in the Testes of Gobiocypris rarus following 17α-Methyltestosterone Exposure
Source: Int J Mol Sci. 2023 Feb 20;24(4):4239. doi: 10.3390/ijms24044239 (PMC9968023; doi:10.3390/ijms24044239)
Supplement: Supplementary file 1 [file ijms-24-04239-s001.zip › Table S8.pdf]

Table S8 Correlations between miRNAs and mRNAs[a].

	miR-122-x	miR-574-x	miR-430-y	miR-217-x	lin-4-x	miR-7-y
faxdc2	0.831	-0.876	0.602	0.268	-0.302	-0.968
inhbb	0.995	0.996	0.640	0.711	-0.704	-0.897
ihhb	0.927	0.914	0.679	0.467	-0.964	-0.897
soat2	0.987	0.984	0.820	0.871	-0.492	-0.450
gatm	0.998	0.997	0.759	0.819	-0.575	-0.811
ebp	0.861	0.870	0.262	0.354	0.460	0.346
cyp1a1	0.998	0.999	0.673	0.741	0.364	0.354

Analyses were performed with SPSS Statistics 22.0.

[a] Analysis was carried out using Pearson's correlation coeffificient.
