# Supplementary figures and images for: Comparative Analysis of miRNA-mRNA Regulation in the Testes of Gobiocypris rarus following 17α-Methyltestosterone Exposure
Source: Int J Mol Sci. 2023 Feb 20;24(4):4239. doi: 10.3390/ijms24044239 (PMC9968023; doi:10.3390/ijms24044239)

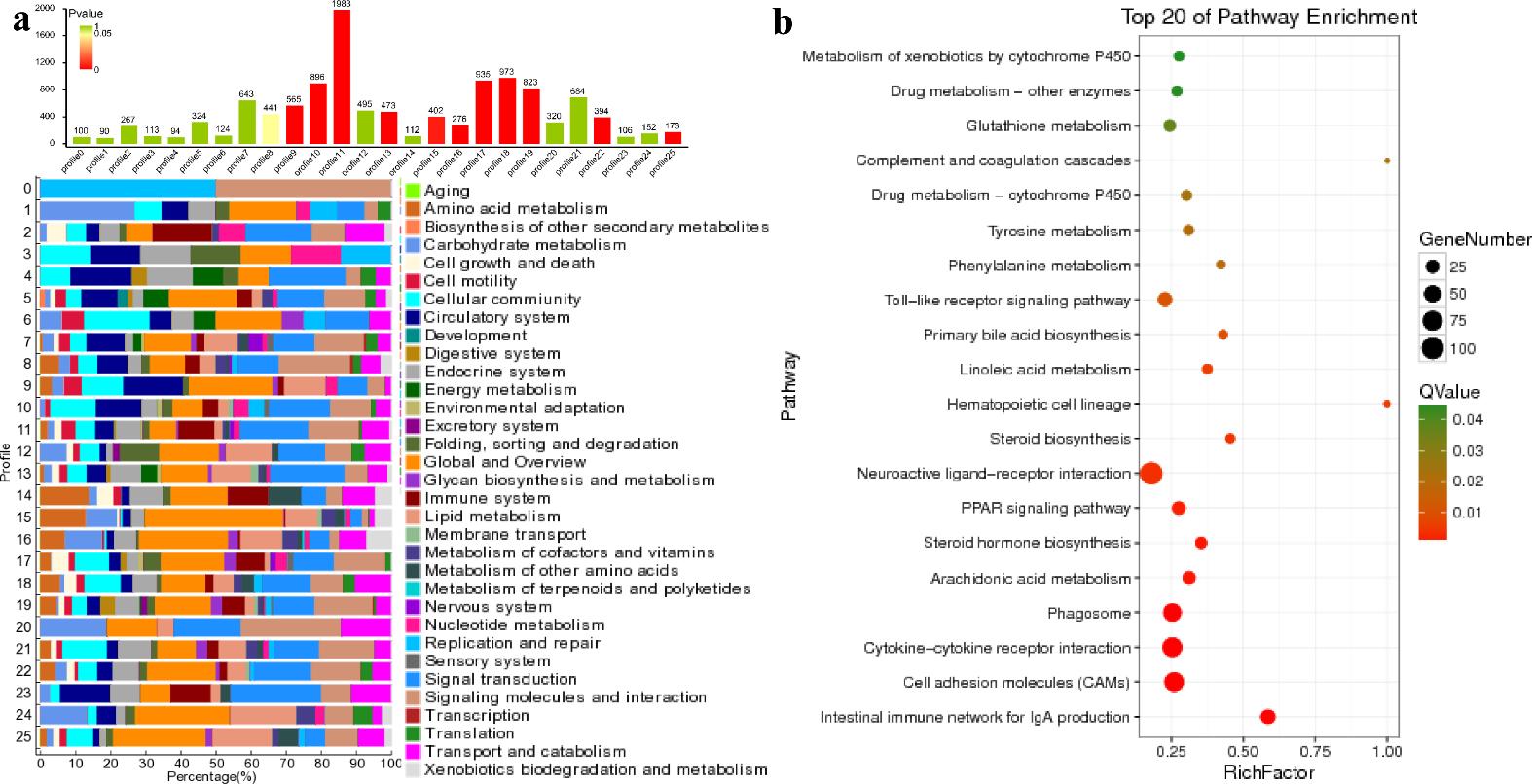

Supplement: Supplementary file 1 [file ijms-24-04239-s001.zip › Figure S1(a,b).jpg]

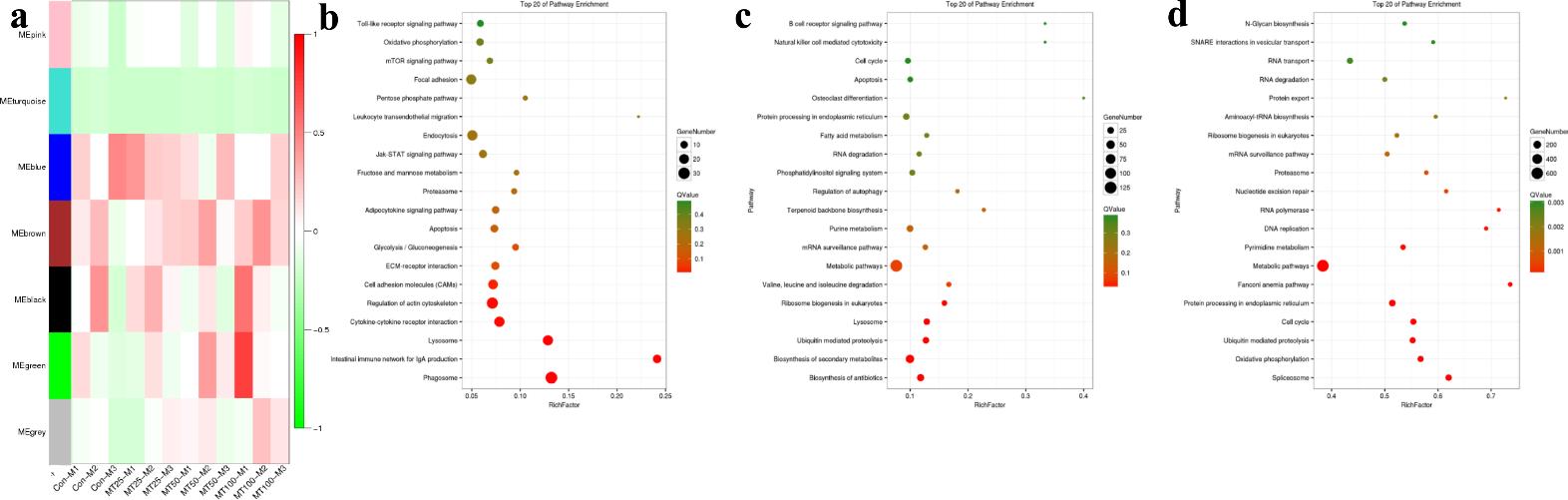

Supplement: Supplementary file 1 [file ijms-24-04239-s001.zip › Figure S2.jpg]

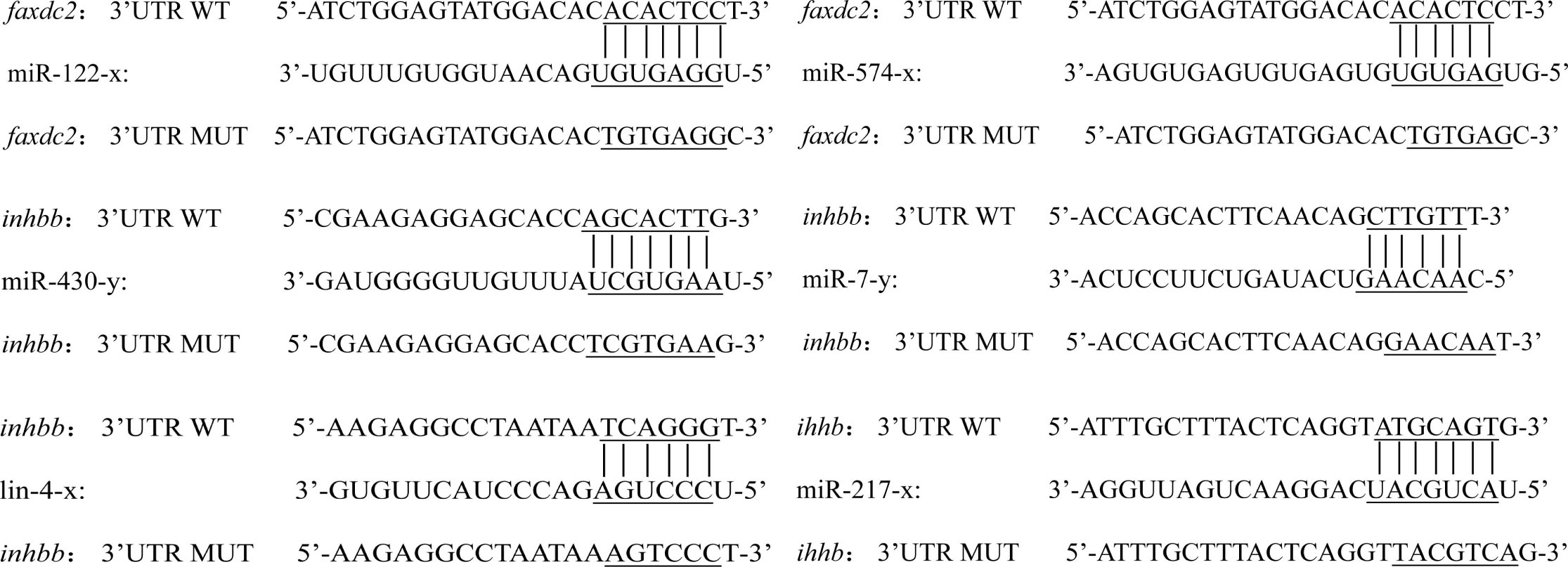

Supplement: Supplementary file 1 [file ijms-24-04239-s001.zip › Figure S3.jpg]
